# Similarity-based pairing improves efficiency of siamese neural networks for regression tasks and uncertainty quantification

**DOI:** 10.1186/s13321-023-00744-6

**Published:** 2023-08-30

**Authors:** Yumeng Zhang, Janosch Menke, Jiazhen He, Eva Nittinger, Christian Tyrchan, Oliver Koch, Hongtao Zhao

**Affiliations:** 1 Medicinal Chemistry, Research and Early Development, Respiratory and Immunology (R&I), BioPharmaceuticals R&D, AstraZeneca, 43183 Gothenburg, Sweden; 2 Department of Pharmaceutical Biosciences, Uppsala University, Uppsala, Sweden; 3 Institute of Pharmaceutical and Medicinal Chemistry, Westfälische Wilhelms-Universität Münster, 48149 Münster, Germany; 4 Molecular AI, Discovery Sciences, R&D, AstraZeneca, 43183 Gothenburg, Sweden

## Abstract

Siamese networks, representing a novel class of neural networks, consist of two identical subnetworks sharing weights but receiving different inputs. Here we present a similarity-based pairing method for generating compound pairs to train Siamese neural networks for regression tasks. In comparison with the conventional exhaustive pairing, it reduces the algorithm complexity from O(n^2^) to O(n). It also results in a better prediction performance consistently on the three physicochemical datasets, using a multilayer perceptron with the circular fingerprint as a proof of concept. We further include into a Siamese neural network the transformer-based Chemformer, which extracts task-specific features from the simplified molecular-input line-entry system representation of compounds. Additionally, we propose a means to measure the prediction uncertainty by utilizing the variance in predictions from a set of reference compounds. Our results demonstrate that the high prediction accuracy correlates with the high confidence. Finally, we investigate implications of the similarity property principle in machine learning.

**Graphical Abstract:**

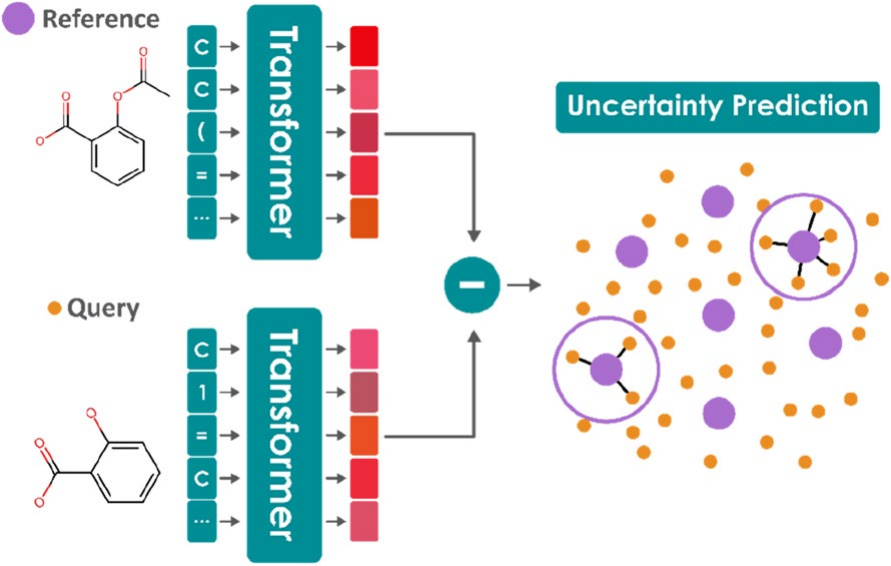

**Supplementary Information:**

The online version contains supplementary material available at 10.1186/s13321-023-00744-6.

## Introduction

Quantitative structure–activity relationship (QSAR) models aim to map from molecular structures to a biological activity or physical property. QSAR models play an essential role in drug discovery, as they allow researchers to quickly gauge crucial properties of molecules without expensive and time-consuming biological experiments [1]. Over the course of the last years deep-learning based models have established themselves as the models of choice. As they provide superior performance on a variety of modeling tasks, including message passing neural networks (MPNN) [2–4], convolutional neural networks (CNN) [5, 6], recurrent neural networks (RNN) [7], and transformers [8–10]. However, such architectures require much data to be trained effectively and such data is not always available in drug discovery. A solution for these low-data regimes could be Siamese neural networks (SNN).

SNN utilizes two identical weights-sharing networks called arms. Both arms receive simultaneously an input. The (dis)similarity of the activations of the two inputs after being propagated is used to train the neural network. This can be done explicitly through specific loss functions like the cosine-loss [11] or the triplet loss [12]. Alternatively, the similarity can be implicitly utilized using the pairwise difference of the activation. The vector of difference is fed into a read-out regression network that predicts the difference in the associated label between the two inputs [13, 14]. For classification, Siamese networks ameliorate the challenging issue of low data; for regression, they can remove the systematic errors associated with a single-arm network by predicting the delta-property, the difference in the property of interest between the two arms. Siamese networks were originally developed in the field of computer vision, for face or handwriting verification [15]. Given its competitive edge in addressing low-data prevailing in drug discovery, it has been applied to the prediction of drug toxicity [16], drug response similarity [17], drug-drug interactions [18], natural product recognition [19], and the classification of bioactivities [7]. Inspired by the relative binding free energy simulation methods which focus on the difference in affinity between two congeneric ligands using a thermodynamic cycle [20], Jimenez-Luna et al. utilized a Siamese convolutional neural network to determine the relative binding affinity between two bound protein − ligand complexes [13]. That seminal work greatly expands the application scope of a Siamese neural network from distance/similarity-based classification to regression. Recently, its prediction performance was further improved by a linear combination of loss terms via the increased regularization of the latent space [14].

A major drawback of Siamese neural networks is the training cost for regression tasks. With an increase in the training data one will observe a combinatorial explosion of the number of pairs as input to the model. Siamese neural networks by design are trained on pairs, which have a complexity of O(n^2^) if all pairs are used for training. It hence becomes computationally expensive to train a deep-learning based Siamese network on a dataset having just a few thousand of compounds as it would result in millions of pairs. However, in the optimization phase of a drug discovery project, medicinal chemists typically make several hundred up to a few thousand of derivatives of a lead compound with small variations, empowered by the high-throughput experimentation [21]. In addition, drug metabolism and pharmacokinetic (DMPK) properties such as aqueous solubility, lipophilicity, and human liver microsome clearance have been routinely measured on newly synthesized compounds, given their important roles in determining the fate of a drug candidate. The DMPK database in a pharmaceutical company could accumulate hundreds of thousands of data points. With exhaustive pairing, it is computationally prohibitive to harness such a wealth of data in a Siamese neural network with affordable resources.

One possible solution is to reduce the number of pairs to train the model. We propose such a strategy using a similarity-based pairing method, inspired by Matched Molecular Pair Analysis (MMPA). MMPA is a cheminformatic method comparing the properties of two molecules that differ only by a single chemical transformation, for example, the substitution of a hydrogen atom by a fluorine one [22, 23]. A matched molecular pair rule for a defined transformation can be derived from the ensemble of corresponding MMPs and their associated property changes. One benefit of MMPA lies on the transferable effect of a chemical transformation, which can then be used to prioritize synthesis [22–26]. Our rationale is that Siamese networks can more easily correlate the structural differences with the property differences when trained on pairs sharing high similarity, which then make it easier to predict the absolute values.

However, not only can we use Siamese networks to make single-point predications for a given molecule, but we can also use it for uncertainty quantification. It has been increasingly recognized as an important aspect in molecular property prediction pipelines, where QSAR models are used to prioritize lab-intensive and time-consuming experimentations [1, 27]. Both the opaque characteristics of deep-learning models and the vast chemical space drive the need for an effective uncertainty quantification [28–30]. Estimates of uncertainty can help users gauge the trustworthiness of the prediction, and point to areas of the chemical space where the model struggles. Popular approaches include ensemble-based methods [31], Bayesian uncertainty estimation [32], and distance-based methods [33]. We propose a method to quantify uncertainties using the variance in predictions from a set of reference compounds.

In this proof-of-concept study we compare the performance of Siamese networks trained with exhaustive pairing versus those trained with the proposed similarity-based pairing strategy. We do this for both a transformer-based model using SMILES strings as input as well as a simple MLP with the circular fingerprints. In addition, we evaluate Siamese networks for uncertainty quantification.

## Methods

### Overview of models

**Fig. 1:**
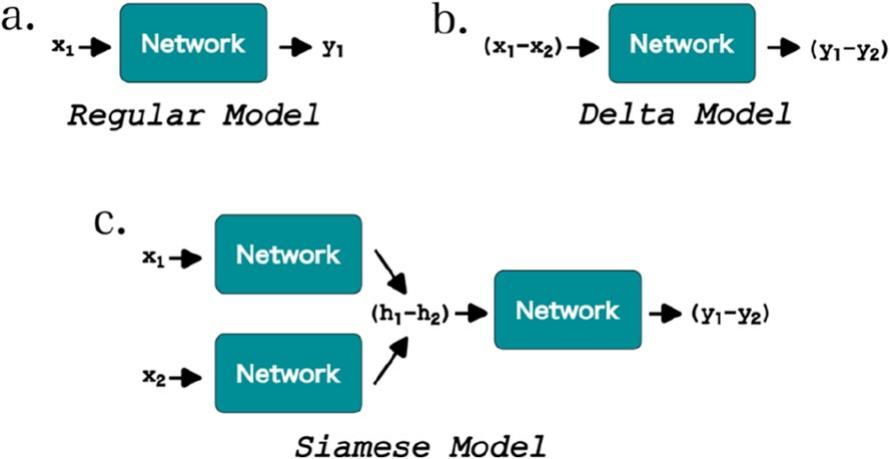
Illustration of the three model designs. **a** The traditional model aims to predict the true value based on a single input. **b** In the delta (Δ) model the difference vector between two samples is used as input and the model predicts their value difference. **c** The Siamese model makes use of the two networks of sharing weights, and the difference between the hidden states is fed through an additional network that predicts the value difference of the two samples

To evaluate whether the proposed similarity-based pairing could perform comparably to exhaustive pairing, we train a variety of models in three designs. The first is the regular model, where the network is fed with a single input instance and is trained to predict the true value of the target variable. This is how traditional neural networks and other statistical models are trained and used. A second design is the delta (Δ) model, where the input vectors of two samples are subtracted from each other, producing a vector of differences. This vector is then used to predict the difference in the target value between the two samples. In the Siamese design, the two samples are separately parsed through a network from which hidden states are derived. Important to note is that the two networks through which the two inputs are parsed have identical weights. The hidden states are subtracted from each other, yielding a vector of differences. This vector is fed into a read-out network which predicts the difference between the two samples. The Siamese neural network is trained, like other models, end-to-end. An overview of the three designs is illustrated in Fig. 1.

### Multilayer perceptron (MLP)

MLP consists of linear transformations and non-linear activation functions. As input to the network, we use the count-based ECFP4 fingerprint, which is rich in chemical information and has been widely used in the field of cheminformatics, including property predictions. In the regular model a chemical property is predicted directly based on the ECFP4. In the delta-variant (called MLP-ΔFP) we subtract from the ECFP4 of a molecule of interest the ECFP4 of a reference molecule with known property. The resulting vector can be viewed as representing the structural difference between the two molecules. The MLP used here have an input layer of 2048 neurons, a hidden layer of 128 neurons followed by a ReLU activation function, and an output layer of a single neuron (Table 1). In the Siamese MLP (MLP-SNN) the two fingerprints are fed in parallel through an MLP. The transformed vectors are subtracted and the resulting difference vector is fed into a read-out layer (Table 1).

**Table 1:** Overview of hyperparameters used

Model	Structure	Max LR	Min LR	Patience	Epsilon	Epochs
MLP	[2048,128,1]	5e-4	1e-5	50	1e-6	70
MLP-ΔFP^*a*^	[2048,128,1]	1e-3	1e-5	50	1e-6	200/150/100
MLP-ΔFP^*b*^	[2048,128,1]	1e-4	1e-6	200	1e-7	100
MLP-SNN^*a*^	[2048,512,128;128,1]	1e-3	1e-5	50	1e-6	200,150,100
MLP-SNN^*b*^	[2048,512,128;128,1]	1e-4	1e-6	200	1e-7	20/40/30
Chemformer	[Dimension = 512, Attention Heads = 8, Layers = 6] [512,64,1]	5e-4	5e-4	0	1e-8	150

^*a*^ trained with the similarity-based pairing and ^*b*^ with exhaustive pairing

### Chemformer

**Fig. 2:**
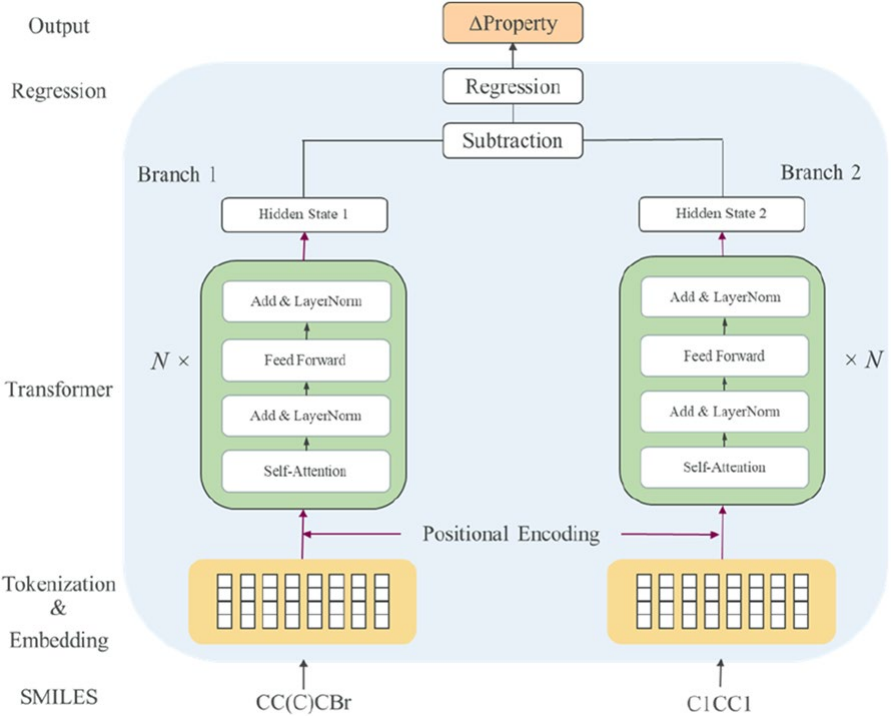
Illustration of the transformer-based Siamese neural network predicting delta-properties

In addition to MLP, we investigate the use of a transformer-based architecture called Chemformer [9], which can handle string-based inputs. SMILES strings are tokenized and embedded with the positional encoding. The encoding layer consists of a self-attention block, an add-layer normalization block, a feedforward block and a second add-layer normalization block. Specifically, the Chemformer uses 6 encoding layers each having 8 attention heads, a model dimension of 512 and a feedforward dimension of 2048 (Table 1). As we cannot subtract SMILES strings from each other the delta-variant of the Chemformer is not trained. In the Siamese design (Fig. 2), each SMILES string of a compound pair is fed into a transformer encoder sharing identical weights. The hidden state of the start token is subtracted from each other, and the subtraction is fed into a read-out regression network, which outputs the delta-property of the compound pair. The model, Chemformer-SNN, was trained with a learning rate of 0.0005 for 150 epochs on the training set, and the state yielding the best performance on the validation set was used to predict the test set. Data augmentation, including both mask and random SMILES strings, was applied during training.

### Random forest with pairwise difference input

In addition, a random forest (RF) model was built with the pairwise difference input, called RF-ΔFP in comparison with the conventional RF-FP, using the default parameters in the Python library scikit-learn.

### Similarity-Based pairing

**Fig. 3:**
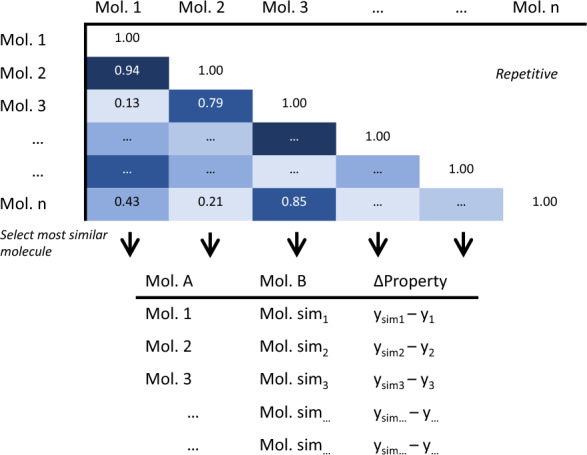
Illustration of the similarity-based pairing. The pair of the highest similarity per column in the lower triangle of the similarity matrix is taken to train a Siamese neural network

The Tanimoto similarity between the two paired compounds was calculated using the open-source cheminformatic tool RDKit (https://www.rdkit.org) with the count-based extended-connectivity fingerprint (ECFP4) [34]. As illustrated in Fig. 3, only the compound pair which has the highest similarity per column in the lower triangle of the similarity matrix was taken to train a Siamese neural network. This results in *N* pairs, in contrast to the *N*^2^/2 pairs from exhaustive pairing [13, 14].

### Random pairing

For the MLP-SNN, it is feasible to compare the performance by the similarity-based pairing with that by exhaustive pairing, however, it is computationally prohibitive for the much larger Chemformer having 45 million parameters. Alternatively, for the Chemformer-SNN, we use a random pairing method. Each compound was paired with 50 randomly selected compounds, as a surrogate of exhaustive pairing.

### Inference of the absolute properties and uncertainty quantification

Each compound in the test set is paired with each compound in the training set, and the delta-properties of the resulting pairs were predicted by the Siamese neural network. Since the compound from the training set of the pair has its property known, the property of the test compound can be determined by Eq. 1:

1 $${\text{Property}}_{{{\text{test}}}} {\text{ = Property}}_{{{\text{training}}}} { + }\Delta {\text{Property}}$$

Each compound in the training set gives rise to a prediction for a test compound. The mean value of all predictions provides a single estimate for the test compound, and the standard deviation provides a way to quantify the prediction uncertainty. The uncertainty quantification by use of Siamese networks is computationally more efficient than ensemble-based approaches, which require multiple networks to be trained with different initializations. To visualize the uncertainty, the confidence curve plotting is adopted, which displays how the error varies with the sequential removal of compounds from the lowest to the highest confidence [28].

We apply the *n*-reference strategy, where *n* is the number of compounds in the training set which share the highest similarity to the test compound. In addition, we introduce a similarity cutoff for choosing reference compounds. Only compounds in the training set having a similarity to a test compound no less than the given cutoff will be chosen as reference. If all compounds in the training set have a similarity below the cutoff to the test compound, that test compound will be excluded from the evaluation. We consider cutoff values ranging from 0.3 to 0.5 with an interval of 0.05.

### Performance metrics

The prediction performance was measured by the pooled root mean square errors (RMSE) and the correlation coefficient *r*^2^ from a tenfold stratified cross-validation.

### Data preparation

The three physiochemical datasets, namely lipophilicity (i.e., logD), freesolv (free energy of solvation) and ESOL (aqueous solubility), were downloaded from Molecule Net [35]. These three datasets have been widely benchmarked against a variety of machine learning models and are of general interest to the community of medicinal chemistry in drug discovery (Table 2). Each of the three datasets was randomly split into a training, a validation and a test set by 80:10:10 with a tenfold stratification. Distribution of the training, the validation and the test set from a single split by t-distributed stochastic neighbor embedding (t-SNE) is shown in Additional file 1: Figure S1. Opposite to the additivity principle which underlies the SAR analysis is nonadditivity (NA), where the combination of two R-groups gives a very different result than the sum of each individual contribution. Nonadditivity presents a great challenge for the QSAR modeling and can be calculated from double-transformation cycles consisting of four compounds that connected by two identical transformations [36]. The amount of nonadditive compounds in each dataset is summarized in Table 2 together with the estimated experimental uncertainty, based on the non-additivity analysis proposed by Kramer [36].

**Table 2:** Summary of the three physicochemical datasets

Property	Lipophilicity	Freesolv	ESOL
Data set size	4200	642	1128
Mean property value	2.19	− 3.80	− 3.05
Standard deviation	1.20	3.85	2.10
Estimated experimental uncertainty (*σ*)	0.2	0.3	0.3
Double transformation cycles	169	7389	8731
Cycles with significant NA (> 2*σ*)	40 (23.7%)	2241 (30.3%)	2660 (30.5%)
Compounds with significant NA (> 2*σ*)	83 (2.0%)	99 (15.4%)	94 (8.4%)
Compounds with strong NA (> 4*σ*)	26 (0.6%)	29 (4.5%)	13 (1.2%)

## Results and discussion

### Similarity-Based pairing outperforms exhaustive pairing

**Fig. 4:**
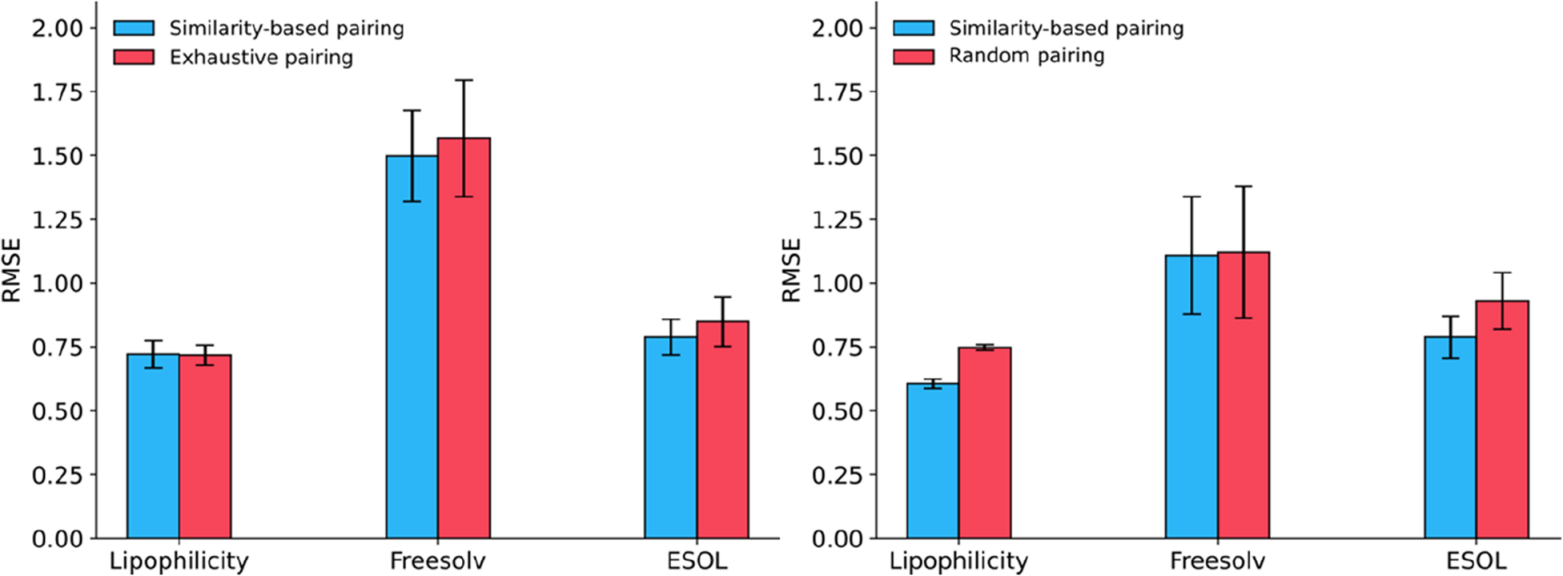
Comparison of the similarity-based pairing with exhaustive pairing to train the MLP-SNN (left), and Chemformer-SNN (right). The number of reference compounds was chosen based on the smallest RMSE as 10 for the lipophilicity, 6 for the freesolv and 7 for the ESOL dataset for the similarity-based pairing, and 8 for the lipophilicity, 10 for the freesolv and 19 for ESOL for exhaustive pairing. For the Chemformer-SNN it was 7, 11 and 7. The error bar indicates the standard deviation from the tenfold cross validation. For the random pairing, each compound was paired with 50 randomly selected compounds as a surrogate of exhaustive pairing to the Chemformer-SNN

**Fig. 5:**
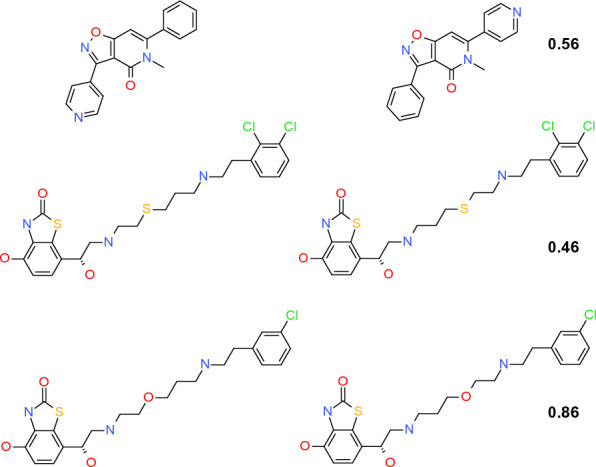
Examples of paired compounds indistinguishable by the ECFP4 fingerprint with the number indicating ΔlogD

The average performance of the different models are shown in Tables 3 and 4. The Siamese networks trained with the similarity-based pairing outperform those trained with exhaustive pairing, consistently across the three datasets and models though the differences are not in itself large (Fig. 4). Not surprisingly the transformer-based models outperform the MLP models. One reason is that the predefined ECFP4 fingerprint, though rich in chemical information, is not task-specific, and in some cases, fails to distinguish the difference between paired compounds (Fig. 5). Transformer-based models have proven to outperform the ECFP4 fingerprint by extracting task-specific features from the SMILES strings only [7, 37, 38]. The performance of the Chemformer-SNN is comparable to that of the Chemformer on the lipophilicity and freesolv datasets, and slightly worse on the ESOL dataset. The slight deteriorating performance of the Chemformer-SNN may arise from the altering Siamese network architecture so that the three datasets are not big enough to fine tune the pretrained Chemformer.

**Table 3:** Performance (RMSE) of pairing strategies

Model	Lipophilicity	Freesolv	ESOL
MLP	0.75	1.60	0.84
MLP-ΔFP	0.74^*a*^ (0.77)^*b*^	1.60^*a*^ (1.66)^*b*^	0.81^*a*^ (0.87)^*b*^
MLP-SNN	0.72^*a*^ (0.72)^*b*^	1.50^*a*^ (1.57)^*b*^	0.79^*a*^ (0.85)^*b*^
RF-FP	0.77	1.91	0.92
RF-ΔFP	0.74^*a*^	1.62^*a*^	0.83^*a*^
Chemformer	0.58	1.07	0.58
Chemformer-SNN	0.61^*a*^ (0.75)^c^	1.11^*a*^ (1.12)^*c*^	0.79^*a*^ (0.93)^*c*^
MolBERT [39]	0.60	1.52	0.55

^*a*^ Trained with the similarity-based pairing

^*b*^ Trained with exhaustive pairing

^*c*^ Trained with the random paring where each compound was paired with 50 randomly chosen compounds as an approximation of exhaustive pairing

**Table 4:** Performance (*r*^2^) of pairing strategies

Model	Lipophilicity	Freesolv	ESOL
MLP-FP	0.61	0.82	0.84
MLP-ΔFP	0.62^*a*^ (0.59)^*b*^	0.82^*a*^ (0.81)^*b*^	0.85^*a*^ (0.82)^*b*^
MLP-SNN	0.64^*a*^ (0.64)^*b*^	0.84^*a*^ (0.83)^*b*^	0.86^*a*^ (0.83)^*b*^
RF-FP	0.58	0.75	0.81
RF-ΔFP	0.62	0.81^*a*^	0.84^*a*^
Chemformer	0.76	0.91	0.92
Chemformer-SNN	0.74^a^ (0.62)^c^	0.91^*a*^ (0.90)^*c*^	0.86^*a*^ (0.80)^*c*^

^*a*^ Trained with the similarity-based pairing

^*b*^ Trained with exhaustive pairing

^*c*^ Trained with the random paring where each compound was paired with 50 randomly chosen compounds as an approximation of exhaustive pairing

**Fig. 6:**
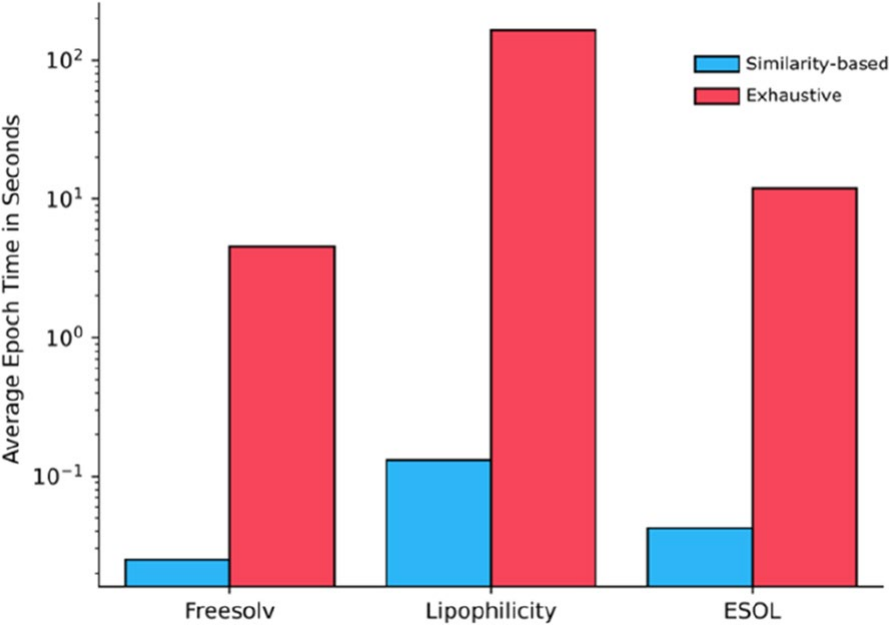
Average trainings time per epoch for the MLP-SNN in seconds on the log scale. Training was done on an Nvidia A40

For the MLP-based models, the MLP-SNN model performs slightly better than the delta and the regular variant. In addition, the RF-ΔFP outperforms the regular RF model. Together, they suggest the potential benefit of using the pairwise difference input in training a machine learning model. In terms of the time to train Siamese neural networks, the similarity-based pairing is several orders of magnitude faster than exhaustive pairing (Fig. 6).

### Increased similarity leads to accurate predictions

**Fig. 7:**
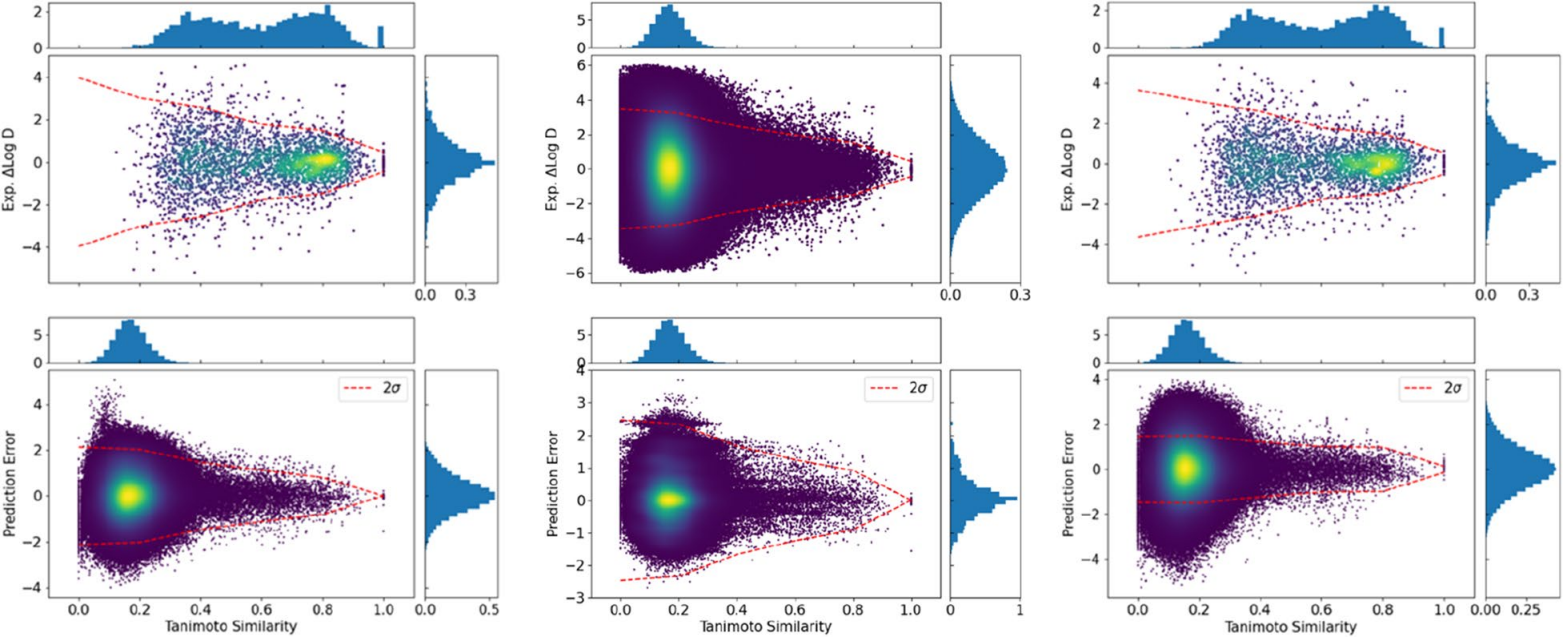
Correlation of the experimental ΔlogD with the Tanimoto similarity for compound pairs in the training set from a single split of the lipophilicity dataset (Top). Correlation of the prediction errors with the Tanimoto similarity for pairs between the test and the training set for the SNN models (Bottom). The left panel refers to the MLP-SNN trained with the similarity-based pairing, the middle to the MLP-SNN with exhaustive pairing, and the right to the Chemformer-SNN with the similarity-based pairing. The red dashed lines indicate the 95% percentile of the distribution

As illustrated in Fig. 7 on the lipophilicity dataset, the similarity-based pairing (top diagram in the left panel) gives rise to pairs having a similarity in the range from 0.2 to 1.0 with two peaks, one at 0.8 and another at 0.4, respectively. The similarity values of the resulting pairs were relatively evenly distributed in the range from 0.3 to 0.8. The ΔlogD of the resulting pairs shows a normal distribution centered at 0 ranging from -4 to 4. The data points at the similarity value of 1 mainly correspond to stereoisomers, and occasionally, the two paired compounds are indistinguishable by the ECFP4 fingerprint (Fig. 5). In sharp contrast, the exhaustive pairing (top diagram in the middle panel) results in a normal distribution of the similarity values centered at around 0.2. Pairs with similarity around 0.2 differ in logD by up to 6 log units, and in comparison, pairs with similarity higher than 0.4 differ by up to only 2 log units.

The similarity property principle in cheminformatics states that compounds with similar chemical structures tend to have similar properties [40]. There, indeed, exists a rather weak trend that the distribution of the experimental ΔlogD shrinks with an increase in the similarity of the compound pair. To have a better understanding of similarity on the prediction accuracy, each compound in the test set was paired with each compound in the training set, and the pairwise property difference was then predicted by the MLP-SNN or Chemformer-SNN model. The prediction error from each pair was measured against the similarity of the two compounds in that pair (the bottom diagram in Fig. 7). Notably, it becomes more pronounced that the prediction error is smaller when the reference compound (i.e., the compound from the training set) is more similar to the test compound, as is evident from the lines depicting the 95% percentile of the distribution. This trend is the same for models trained through both exhaustive pairing and similarity-based pairing. It thus indicates that the relationship between prediction accuracy and similarity to reference compounds does not arise from the similarity-based pairing, but rather is a general property.

For many physicochemical properties such as logD, a single heavy atom change by an ionizable amine or an alcohol could drastically alter the property despite the resulting compound being similar to parent ones, giving rise to the property cliffs manifested by the large property difference between two structurally similar compounds. As shown in Fig. 7, some pairs of similarity greater than 0.8 have the experimental ΔlogD around 3, suggesting they form property cliffs. However, such effects are arguably transferable and hence predictable, underlying the concept of matched molecular pair analysis in medicinal chemistry [22, 23]. The capability of predicting property cliffs by Siamese networks is implied by the lack of significant outliers in the prediction errors at the similarity higher than 0.8. The analysis on the other two datasets reveals qualitatively similar observations (Additional file 1: Figure S2 and S3).

### Effect of the number of reference compounds

**Fig. 8:**
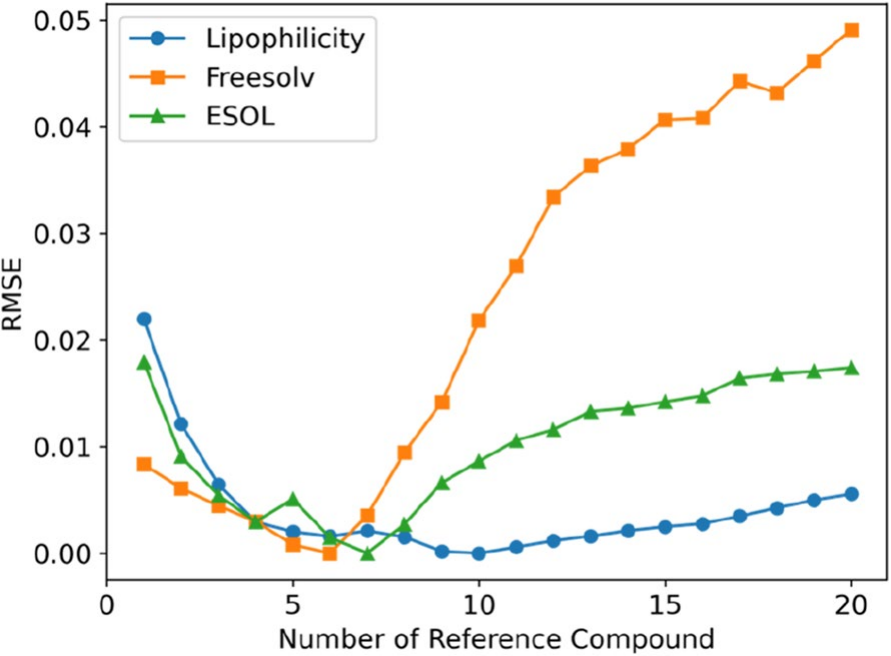
Performance dependence on the number of reference compounds for the MLP-SNN models. For an easy comparison, the global minimum of each curve was shifted to 0 by 0.72 at *n* = 10 for the lipophilicity, 1.50 at *n* = 6 for the freesolv, and 0.79 at *n* = 7 for the ESOL dataset

Given the observation that reference compounds similar to a test compound yield a small prediction error, we investigate the impact of the number of reference compounds on the prediction accuracy. The compounds in the training set were ranked by their similarities to a test compound, and the top *n* compounds were chosen as reference to infer the absolute property of the test compound. Notably, the one-reference learning does not give rise to the lowest RMSE in comparison with the ensemble-based learning, although the single reference is most similar to the test compound (Fig. 8). As the number of references compounds increases, an initial improvement in performance can be observed. However, at a specific point this trend shifts and the performance starts to degrade by adding more reference compounds. A possible explanation for this behavior is that with very few reference compounds the bias of the reference compounds might weigh too much, particularly in the case of activity/property cliffs. On the other hand, having too many reference compounds, it leads to a scenario where some of the reference compounds are not similar enough to the test molecule and hence not predictive.

**Fig. 9:**
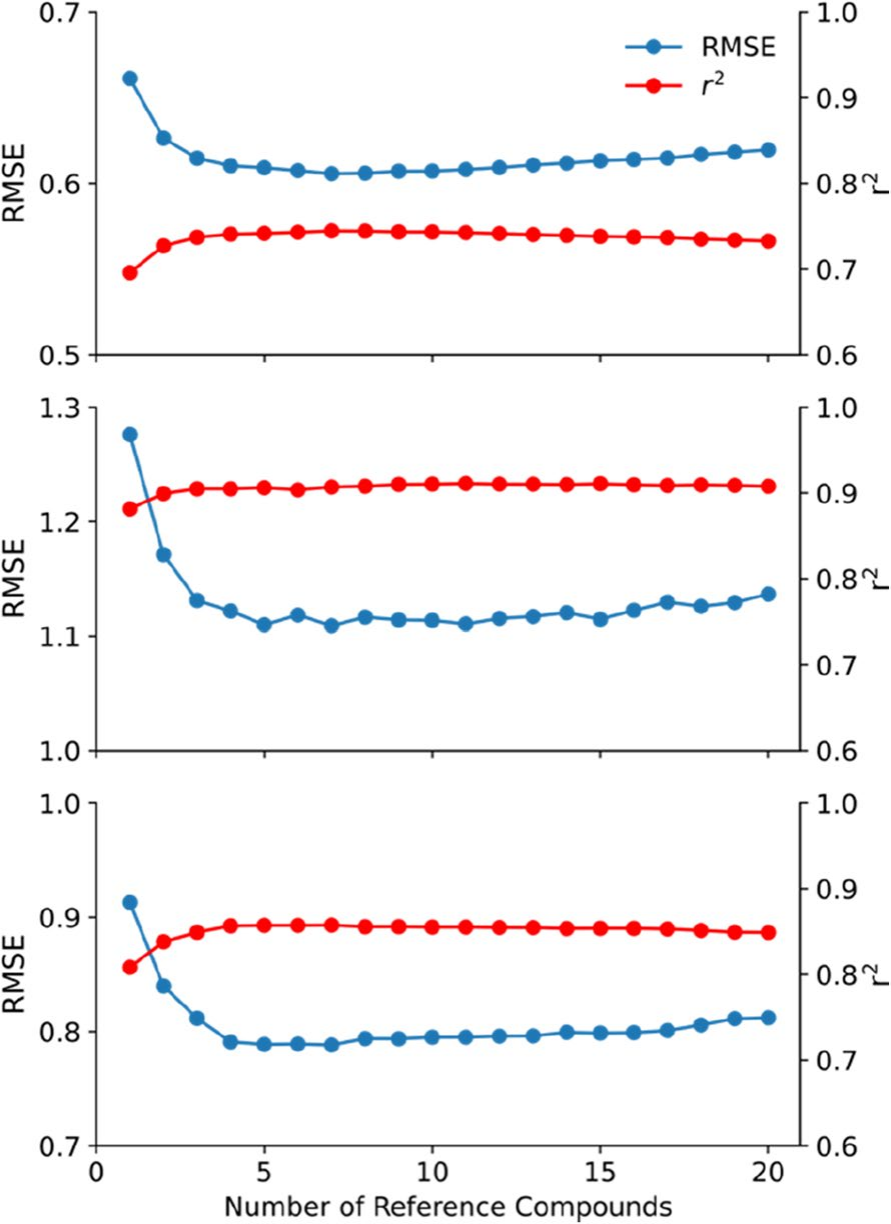
Performance dependence on the number of reference compounds for the Chemformer-SNN from the tenfold stratified cross-validation on the lipophilicity (Top), freesolv (Middle) and ESOL dataset (Bottom)

The prediction performance of the Chemformer-SNN becomes stable on all three datasets after five reference compounds (Fig. 9). In comparison with the MLP-SNN, there is no significant deterioration in the prediction performance with an increase in the number of reference compounds, up to 20.

### Uncertainty quantification

**Fig. 10:**
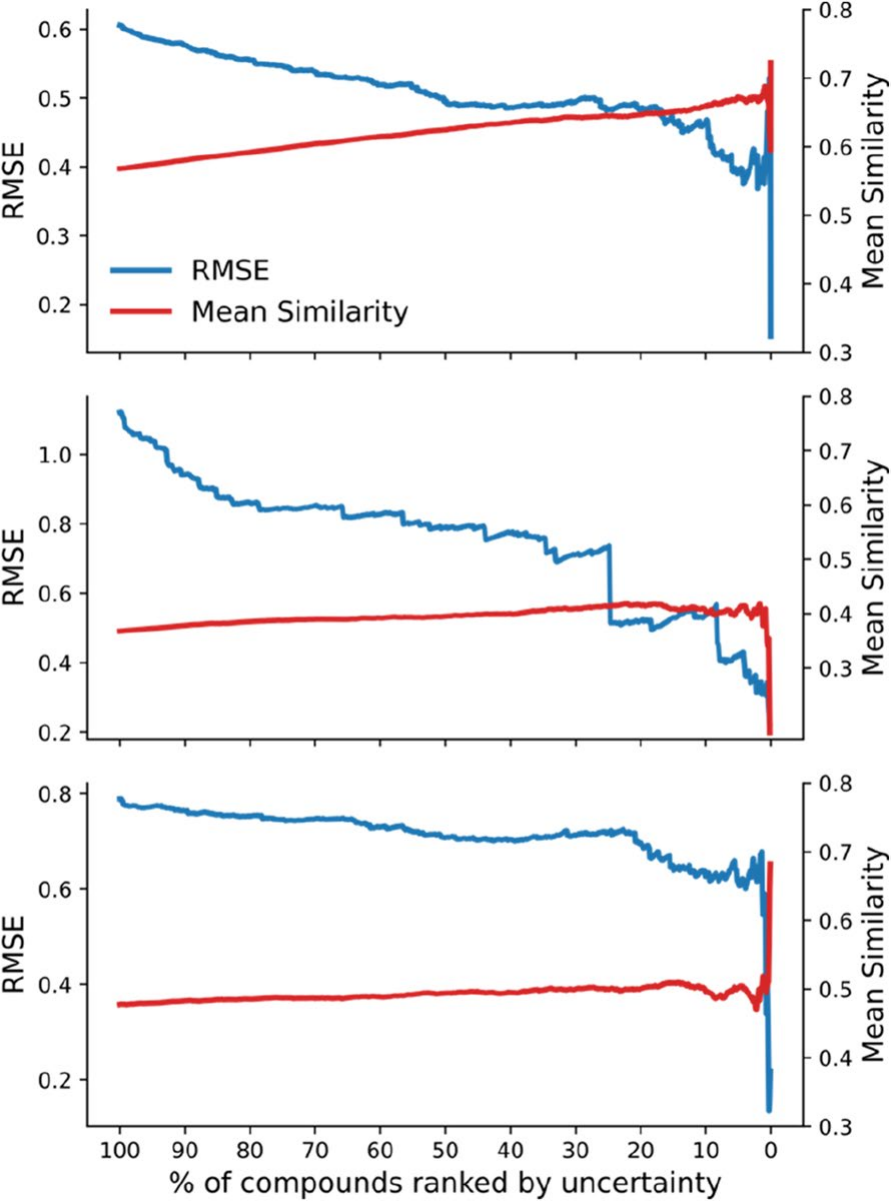
Quantification of uncertainties in the predictions by the Chemformer-SNN for the lipophilicity (Top), the freesolv (Middle) and the ESOL dataset (Bottom)

To visualize the uncertainty, the confidence curve plotting is adopted, which displays how the error varies with the sequential removal of compounds from the lowest to the highest confidence [28]. As shown in Fig. 10, the prediction error of RMSE decreases on all three datasets when compounds with low confidence are sequentially removed. The relationship between the high confidence and small prediction error is evident. For example, removal of the 20% compounds with the highest uncertainty decreases the RMSE from 1.1 to 0.7 on the freesolv dataset. Concomitantly, the average similarity of reference compounds to test compounds corresponds with the increase in confidence, in line with the similarity principle. Intriguingly, when less than 10% compounds were left, fluctuations in RMSE were observed. This could be ascribed to the statistical noise due to an insufficient number of compounds in the evaluation of RMSE, which could be affected by activity cliffs [41, 42] or nonadditivity [43].

**Fig. 11:**
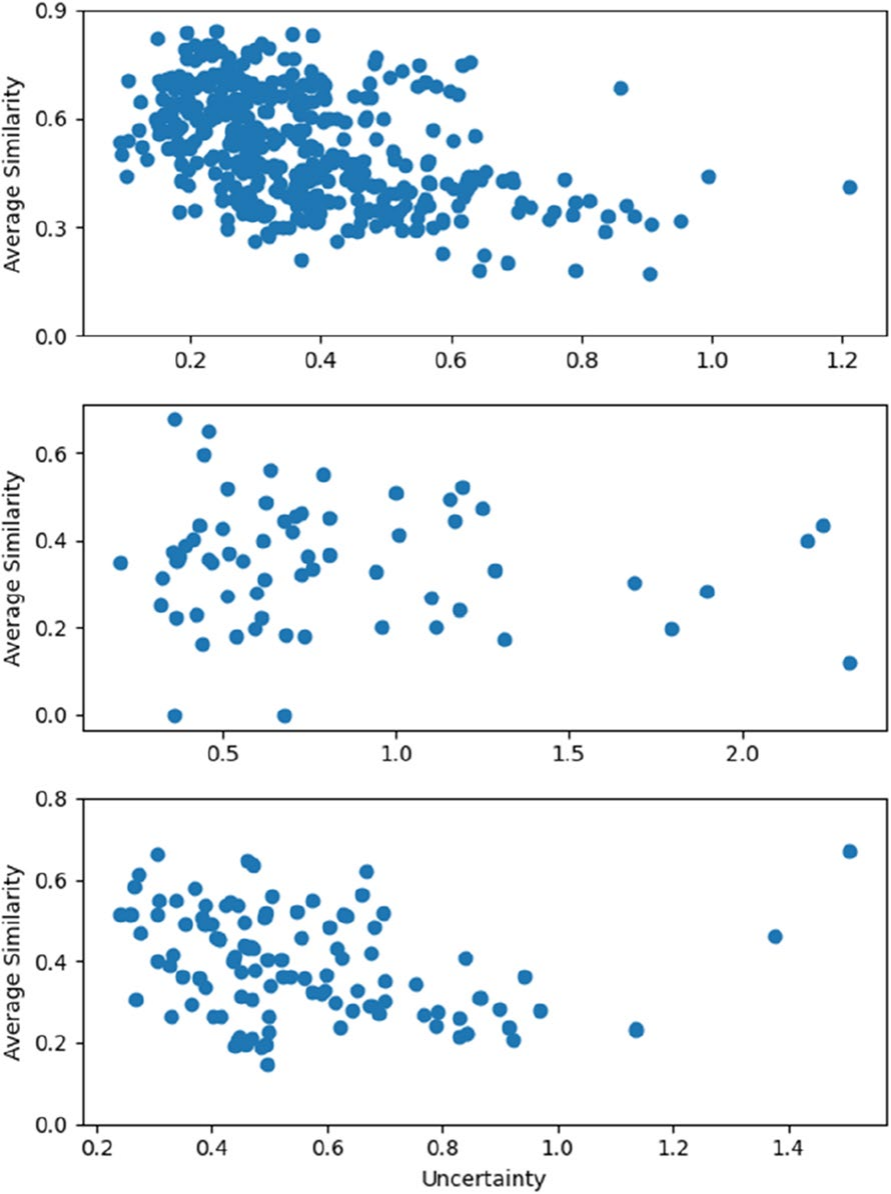
Correlation of uncertainty with the average similarity of reference compounds by the 10-reference learning on the lipophilicity (Top), the freesolv (Middle) and the ESOL dataset (Bottom) from a single split

The detailed view of the correlation of uncertainty with the average similarity of reference compounds reveals a general trend that the uncertainty increases with the decrease in similarity, most prominent on the lipophilicity dataset (Fig. 11). However, outliers do exist. High uncertainty at high similarity could be an indication of activity cliffs or non-linear SAR contributions (e.g., the non-additivity from double-transformation cycles). Intriguingly, low uncertainty at low similarity has been observed too.

### Implications of the similarity principle

**Fig. 12:**
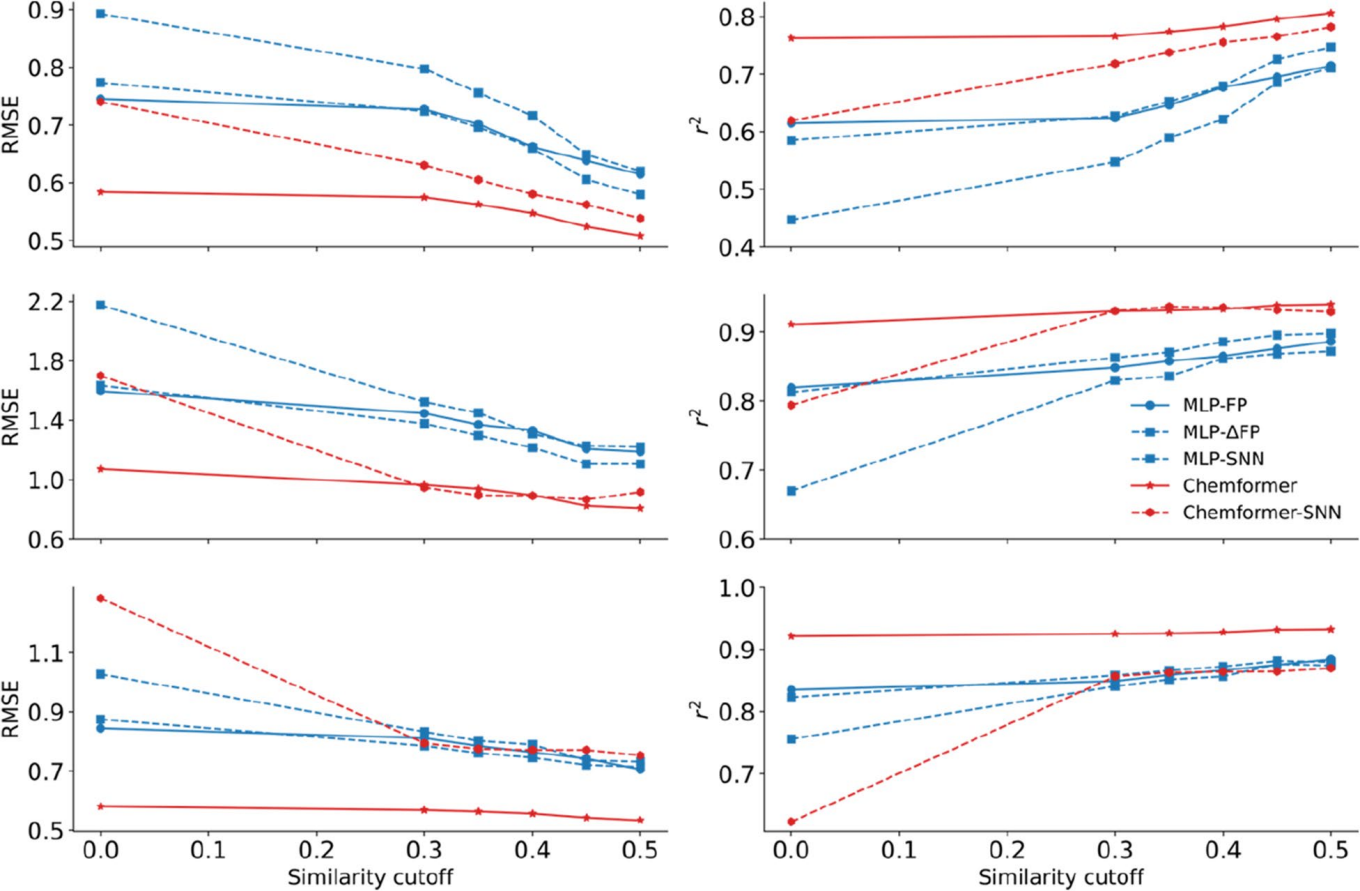
Performance dependence on the cutoff of the Tanimoto similarity between the test and the training compounds from the tenfold cross validation for the lipophilicity (Top), freesolv (Middle) and ESOL (Bottom) datasets

To further evaluate the impact of the similarity principle on machine learning, we compare the prediction errors at different similarity cutoffs. For all models, if the highest similarity between a test compound and any compound in the training set is less than the given cutoff, that test compound is excluded from the evaluation. This leads to the exclusion of 1.4%, 4.7%, 9.7%, 15.6% and 21.1% of the data at the cutoff of 0.3, 0.35, 0.4, 0.45 and 0.5 for lipophilicity; 5.7%, 9.3%, 16.9%, 23.7% and 33.4% for freesolv; 5.1%, 8.7%, 14.9%, 21.3% and 30.9% for ESOL, respectively. As shown in Fig. 12, the prediction error of RMSE decreases with an increase in the similarity for all models and the correlation coefficient *r*^2^ increases correspondingly, signifying the role of the similarity principle. Our observations corroborate the previous findings that the prediction error associated with a molecule rather depends on its distance to the training molecules [33, 44, 45]. Dependence of the prediction performance on the similarity is striking for both the MLP-SNN and Chemformer-SNN. The similarity-based pairing is designed to capture the transferable effect of a small chemical transformation, inspired by the concept of matched molecular pair analysis. When the two paired compounds are extremely dissimilar to each other, poor predictions can be expected since the transformation now concerns the two molecules, rather than a few local variations.

## Conclusions

In summary, we propose a similarity-based pairing method to generate compound pairs for training a Siamese neural network. Our results show that it performs equivalently with the exhaustive pairing and reduces the model complexity from O(n^2^) to O(n), hence making it tractable to train a deep-learning based Siamese neural network on a big dataset. Combining the Siamese neural network with multiple reference compounds, we further quantify the prediction uncertainty and show that the high prediction accuracy indeed correlates with the high confidence. Therefore, the uncertainty quantification could be used to guide experimental designs by selecting compounds of high uncertainty for exploration and compounds of low uncertainty for exploitation.

### Supplementary Information

**Additional file 1: Figure S1**. t-SNE plot using the ECFP4 fingerprints for the lipophilicity (A), Freesolv (B) and ESOL (C) dataset on the training, validation and test set from a single split. **Figure S2**. Correlation of the experimental delta-property with the Tanimoto similarity for compound pairs in the training set from a single split of the solubility (Top left) and free solvation energy dataset (Top right). Correlation of the prediction errors with the Tanimoto similarity for pairs between the test and the training set for the MLP-SNN model (Bottom). The red dashed lines indicate the 95% percentile of the distribution. **Figure S3**. Correlation of the experimental delta-property with the Tanimoto similarity for compound pairs in the training set from a single split of the solubility (Top left) and free solvation energy dataset (Top right). Correlation of the prediction errors with the Tanimoto similarity for pairs between the test and the training set for the Chemformer-SNN model (Bottom). The red dashed lines indicate the 95% percentile of the distribution.

## Data Availability

The data sets used in this study are available on the MoleculeNet (https://moleculenet.org/). The source code is publicly available on the GitHub https://github.com/AstraZeneca/Siamese-Regression-Pairing.
