# Supplementary material for: Similarity-based pairing improves efficiency of siamese neural networks for regression tasks and uncertainty quantification
Source: J Cheminform. 2023 Aug 30;15:75. doi: 10.1186/s13321-023-00744-6 (PMC10469421; doi:10.1186/s13321-023-00744-6)
Supplement: Supplementary file 1 — Additional file 1: Figure S1. t-SNE plot using the ECFP4 fingerprints for the lipophilicity (A), Freesolv (B) and ESOL (C) dataset on the training, validation and test set from a single split. Figure S2. Correlation of the experimental delta-property with the Tanimoto similarity for compound pairs in the training set from a single split of the solubility (Top left) and free solvation energy dataset (Top right). Correlation of the prediction errors with the Tanimoto similarity for pairs between the test and the training set for the MLP-SNN model (Bottom). The red dashed lines indicate the 95% percentile of the distribution. Figure S3. Correlation of the experimental delta-property with the Tanimoto similarity for compound pairs in the training set from a single split of the solubility (Top left) and free solvation energy dataset (Top right). Correlation of the prediction errors with the Tanimoto similarity for pairs between the test and the training set for the Chemformer-SNN model (Bottom). The red dashed lines indicate the 95% percentile of the distribution. [file 13321_2023_744_MOESM1_ESM.docx]

**Supporting Information**

**Similarity-Based Pairing Improves Efficiency of Siamese Neural Networks for Regression Tasks and Uncertainty Quantification**

Yumeng Zhang,^1,2^ Janosch Menke,^*1,3^ Jiazhen He,^4^ Eva Nittinger,^1^ Christian Tyrchan,^1^ Oliver Koch,^3^ and Hongtao Zhao^1*^

^1^Medicinal Chemistry, Research and Early Development, Respiratory and Immunology (R&I), BioPharmaceuticals R&D, AstraZeneca, Gothenburg 43183, Sweden; ^2^Department of Pharmaceutical Biosciences, Uppsala University, Uppsala, Sweden; ^3^Institute of Pharmaceutical and Medicinal Chemistry, Westfälische Wilhelms-Universität Münster, Münster 48149, Germany; ^4^Molecular AI, Discovery Sciences, R&D, AstraZeneca, Gothenburg 43183, Sweden

________________________________________

^*^Email: janosch@chalmers.se (J. Menke)

hongtao.zhao@astrazeneca.com (H. Zhao)

**Table S1. Summary of different models.**

| **Models** | **Descriptors** | **Input** | **Output** |
| --- | --- | --- | --- |
| MLP-FP | ECFP4 | single | property |
| MLP-ΔFP | ΔECFP4 | pair | Δproperty |
| MLP-SNN | Difference in the encoded molecular representation between a pair of input compounds | pair | Δproperty |
| RF-FP | ECFP4 | single | property |
| RF-ΔFP | ΔECFP4 | pair | Δproperty |
| Chemformer | Encoded molecular representation | single | property |
| Chemformer-SNN | Difference in the encoded molecular representation between a pair of input compounds | pair | Δproperty |

**
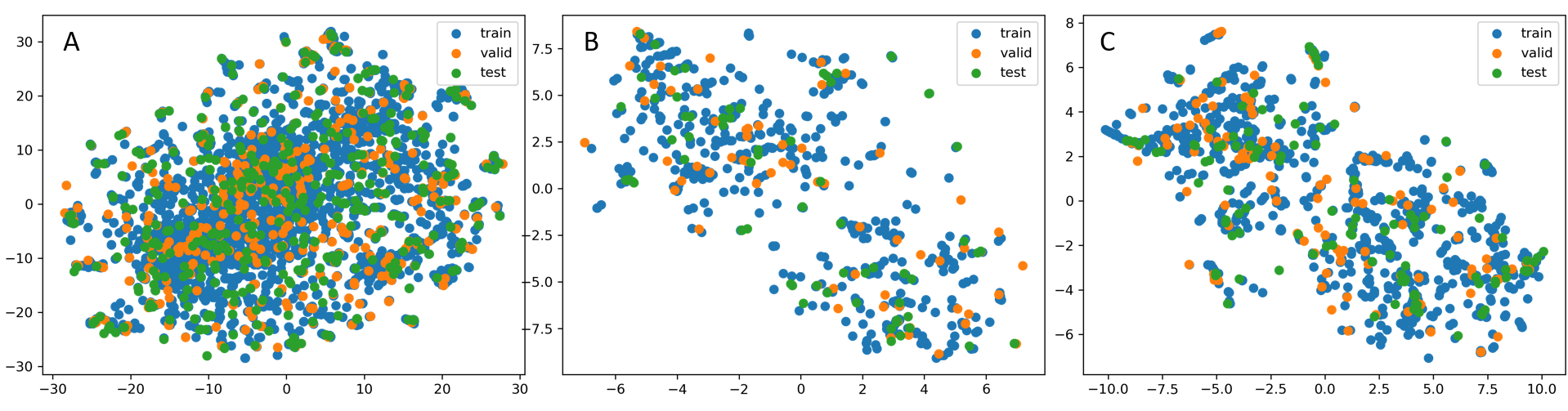
**

**Figure S1.** t-SNE plot using the ECFP4 fingerprints for the lipophilicity (A), Freesolv (B) and ESOL (C) dataset on the training, validation and test set from a single split.


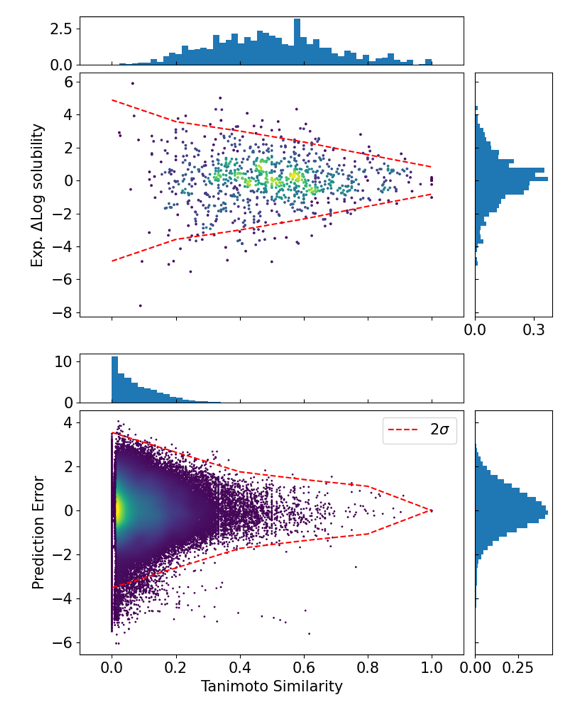

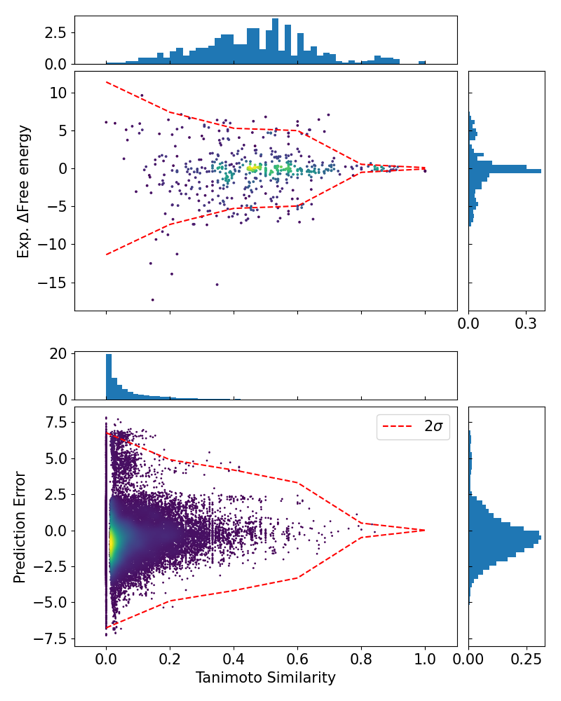


**Figure S2.** Correlation of the experimental delta-property with the Tanimoto similarity for compound pairs in the training set from a single split of the solubility (Top left) and free solvation energy dataset (Top right). Correlation of the prediction errors with the Tanimoto similarity for pairs between the test and the training set for the MLP-SNN model (Bottom). The red dashed lines indicate the 95% percentile of the distribution.


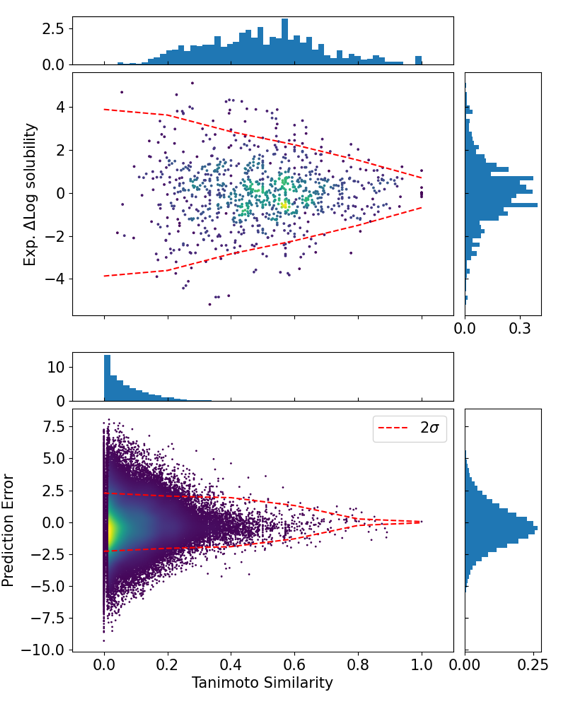

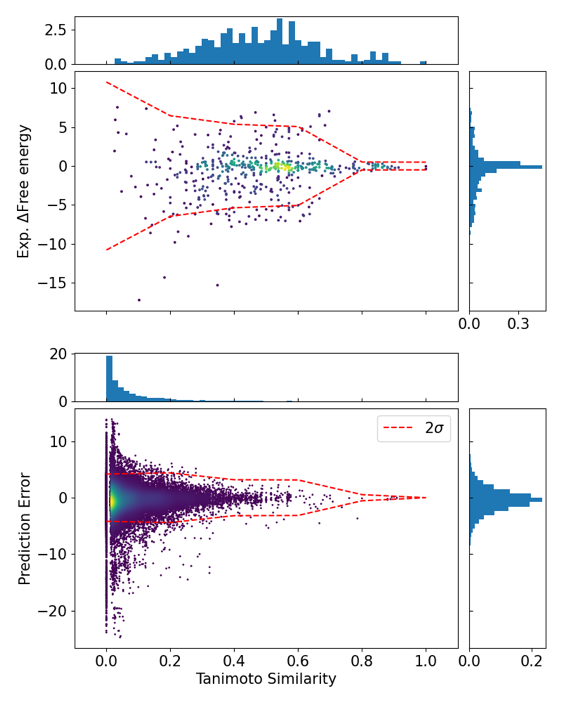


**Figure S3.** Correlation of the experimental delta-property with the Tanimoto similarity for compound pairs in the training set from a single split of the solubility (Top left) and free solvation energy dataset (Top right). Correlation of the prediction errors with the Tanimoto similarity for pairs between the test and the training set for the Chemformer-SNN model (Bottom). The red dashed lines indicate the 95% percentile of the distribution.
